# Association Among Household Water, Sanitation, and Hygiene (WASH) Status and Typhoid Risk in Urban Slums: Prospective Cohort Study in Bangladesh

**DOI:** 10.2196/41207

**Published:** 2023-11-20

**Authors:** Birkneh Tilahun Tadesse, Farhana Khanam, Faisal Ahmmed, Xinxue Liu, Md Taufiqul Islam, Deok Ryun Kim, Sophie SY Kang, Justin Im, Fahima Chowdhury, Tasnuva Ahmed, Asma Binte Aziz, Masuma Hoque, Juyeon Park, Gideok Pak, Hyon Jin Jeon, Khalequ Zaman, Ashraful Islam Khan, Jerome H Kim, Florian Marks, Firdausi Qadri, John D Clemens

**Affiliations:** 1 Epidemiology, Public Health, Impact Unit International Vaccine Institute Seoul Republic of Korea; 2 Infectious Diseases Division International Centre for Diarrhoeal Disease Research Dhaka Bangladesh; 3 Oxford Vaccine Group, Department of Pediatrics University of Oxford Oxford United Kingdom; 4 Cambridge Institute of Therapeutic Immunology and Infectious Disease, University of Cambridge School of Clinical Medicine Cambridge United Kingdom; 5 Madagascar Institute for Vaccine Research University of Antananarivo Antananarivo Madagascar

**Keywords:** water, sanitation, sanitary, contaminated, contamination, hygiene, hygienic, WASH, water, sanitation and hygiene, typhoid fever, enteric fever, typhus, typhoid, enteric, salmonella, protection, recursive partitioning, Bangladesh, low- and middle-income countries, LMIC, bacteria, bacterial, bacterial infection, machine learning, algorithm, algorithms, model, low income, slum, slums, risk, infection control, incidence, prevalence, epidemiology, epidemiological, poverty

## Abstract

**Background:**

Typhoid fever, or enteric fever, is a highly fatal infectious disease that affects over 9 million people worldwide each year, resulting in more than 110,000 deaths. Reduction in the burden of typhoid in low-income countries is crucial for public health and requires the implementation of feasible water, sanitation, and hygiene (WASH) interventions, especially in densely populated urban slums.

**Objective:**

In this study, conducted in Mirpur, Bangladesh, we aimed to assess the association between household WASH status and typhoid risk in a training subpopulation of a large prospective cohort (n=98,087), and to evaluate the performance of a machine learning algorithm in creating a composite WASH variable. Further, we investigated the protection associated with living in households with improved WASH facilities and in clusters with increasing prevalence of such facilities during a 2-year follow-up period.

**Methods:**

We used a machine learning algorithm to create a dichotomous composite variable (“Better” and “Not Better”) based on 3 WASH variables: private toilet facility, safe drinking water source, and presence of water filter. The algorithm was trained using data from the training subpopulation and then validated in a distinct subpopulation (n=65,286) to assess its sensitivity and specificity. Cox regression models were used to evaluate the protective effect of living in “Better” WASH households and in clusters with increasing levels of “Better” WASH prevalence.

**Results:**

We found that residence in households with improved WASH facilities was associated with a 38% reduction in typhoid risk (adjusted hazard ratio=0.62, 95% CI 0.49-0.78; *P*<.001). This reduction was particularly pronounced in individuals younger than 10 years at the first census participation, with an adjusted hazard ratio of 0.49 (95% CI 0.36-0.66; *P*<.001). Furthermore, we observed an inverse relationship between the prevalence of “Better” WASH facilities in clusters and the incidence of typhoid, although this association was not statistically significant in the multivariable model. Specifically, the adjusted hazard of typhoid decreased by 0.996 (95% CI 0.986-1.006) for each percent increase in the prevalence of “Better” WASH in the cluster (*P*=.39).

**Conclusions:**

Our findings demonstrate that existing variations in household WASH are associated with differences in the risk of typhoid in densely populated urban slums. This suggests that attainable improvements in WASH facilities can contribute to enhanced typhoid control, especially in settings where major infrastructural improvements are challenging. These findings underscore the importance of implementing and promoting comprehensive WASH interventions in low-income countries as a means to reduce the burden of typhoid and improve public health outcomes in vulnerable populations.

## Introduction

Typhoid fever, also known as enteric fever, is a deadly febrile illness caused by the *Salmonella enterica* serovar Typhi (*Salmonella* Typhi) [1,2]. Based on 2019 data, approximately 9 million people contract typhoid annually, leading to 110,000 deaths [3]. Common symptoms include persistent fever, fatigue, headache, nausea, abdominal pain, and digestive issues, and occasionally a rash [2,3]. Severe cases of typhoid fever can have life-threatening complications and result in fatality [4]. The effectiveness of treating typhoid has been compromised by the growing resistance to various antibiotics [5,6]. To address this challenge, the World Health Organization advocates for the use of the typhoid conjugate vaccine (TCV) in children aged 6 months and older [3]. Nevertheless, the implementation of vaccination programs alone will not be enough to effectively control and eliminate the transmission of typhoid fever, particularly in urban slum areas where water, sanitation, and hygiene (WASH) facilities and practices are inadequate [7].

The primary mode of transmission for *Salmonella* Typhi is through the fecal-oral route via consumption of food and water that has been contaminated [2]. Consequently, inadequate access to clean water, improved sanitary facilities, appropriate waste management, and proper hand hygiene practices can contribute to an increased risk of typhoid fever [7,8]. For instance, successful elimination of typhoid fever in high-income countries is primarily attributed to major improvements in WASH facilities and practices [7,9]. However, substantial WASH modernization requires major financial, capital, and political commitment, and continues to be an unmet need in resource-constrained settings where unsafe drinking water and lack of sanitary facilities, augmented by unplanned urbanization, significantly contribute to increased typhoid transmission [10,11].

The need for multidimensional interventions, which now include vaccination using available TCVs, to prevent exposure to and transmission of typhoid fever demands implementation of feasible approaches to improving WASH, especially within densely populated urban settings. The emphasis on implementing practical enhancements in WASH is significant, yet remains relevant until a comprehensive upgrade of WASH practices and infrastructure is implemented in settings with high typhoid burden. Addressing the problem of typhoid fever in urban slums is particularly important as the highest burden has been reported in these settings including the occurrence of enhanced environmental transmission [12,13]. For example, in urban slums in Bangladesh, a high burden of typhoid fever (incidence of 200 per 100,000 person years of observation; PYO) and paratyphoid fever (incidence of 40 per 100,000 PYO) was reported [14]. The implementation of WASH practices has the potential to yield critical benefits, including the reduction of *Salmonella* Typhi transmission within communities. By mitigating the prevalence of this bacterium, the burden of bloodstream infections caused by *Salmonella* Typhi can be diminished. As a result, the consumption of antibiotics may be significantly reduced, contributing to the ongoing battle against antimicrobial resistance.

Our earlier work demonstrated that existing improvements in WASH facilities in a Kolkata slum were associated with reduced risk of typhoid (hazard ratio [HR]=0.57, 95% CI 0.37-0.90; *P*=.015) [8]. In this work, we developed a decision tree defining “Good” *versus* “Not Good” household WASH using 4 variables: source of drinking water, hand washing with soap, presence of flush toilet, and source of daily water use. The decision tree successfully discriminated between households at higher versus lower risk of typhoid (area under the curve [AUC] of 58%, 95% CI 54-61; sensitivity of 90.4%, 95% CI 84.2-94.8; and specificity of 19.8%, 95.5 CI 19.3-20.3). Notably, though, the data used for this analysis were accrued more than 20 years ago, possibly limiting their generalizability to circumstances in impoverished urban slums with endemic typhoid today.

Here we investigate whether existing improvements in WASH in a contemporary slum in Dhaka were also associated with a reduced risk of typhoid. We reexamined the hypothesis that variations in household WASH already present in urban slums can successfully predict the risk of typhoid fever. We test this hypothesis using the data from prospective, population-based, surveillance for typhoid fever in the control clusters of a cluster randomized trial of TCV recently completed in urban slums of Mirpur, Dhaka, Bangladesh [15].

## Methods

### Cluster Randomized Trial on the Vi-Tetanus Toxoid Vaccine

A cluster randomized trial (CRT) was conducted between May 2018 and March 2020 with the aim to evaluate the protection, including herd protection, conferred by Vi-tetanus toxoid conjugate vaccine (Vi-TT, Typbar-TCV, Bharat Biotech International Limited) when administered in a mass vaccination campaign. This participant- and observer-blinded CRT included children 9 months to <16 years within a censused total population of 205,760, where 150 clusters were randomized to receive Vi-TT or Japanese encephalitis (JE) vaccines (control clusters) in a 1:1 ratio. These clusters were approximately of comparable geographic size with mean area of 0.02 (SD 0.01) km^2^ and included contiguous households with approximately equal total populations separated by natural borders wherever possible [7]. Age-eligible, nonpregnant residents who provided informed consent received a single dose of the assigned vaccine. The CRT was implemented in the Mirpur area in Bangladesh located in the north-east of Dhaka city. Mirpur is a densely populated settlement with a total surface area of 58.66 km^2^ and approximately 3.5 million residents, serving as a field study site since 1987.

A baseline census was performed between February 14 and March 25, 2018, to enumerate the entire population in this study’s area, except nonpermanent residents and individuals who planned to move out within a month. During the census, individual- and household-level socioeconomic and demographic data, details on water-sanitation-hygiene facilities and practices, and geo-positioning coordinates of each household were collected after obtaining informed consent. A “household” was defined as persons sharing the same cooking pot. The census was subsequently updated at approximately 6-month intervals to capture all births, deaths, and migrations. At each census, 13 nonbinary variables characterizing household WASH were collected. Because there were no significant differences between the household WASH status at first census and the subsequent updates by household, WASH characteristics at first census participation were used for this analysis. Accordingly, WASH variables collected during the baseline census were used to characterize household WASH status for individuals present in this study’s area during the baseline census; the date of participation in the baseline census was taken as the start date of follow-up. For individuals who moved into the clusters after the baseline census and for births, the WASH variables collected during the first census update after the onset of their first participation were used to characterize household WASH status. The date of migration in and the date of birth were considered as the starting date of follow-up of these participants.

### Typhoid Fever Surveillance

Surveillance for typhoid fever started on February 26, 2018, in all age groups using a common protocol across all 8 clinical facilities (Mirpur Field Clinic; Suhrawardy Hospital; Radda Barnen; Adhunik Hospital; Shishu hospital; Kurmitola General Hospital; Kingston Hospital; Islami Bank Hospital) serving this study’s population. Biweekly home visits of the entire population were performed by community health workers to promote care seeking of all household members for febrile illnesses at one of this study’s facilities. Blood samples for microbiological culture (3 mL for ≤17 years, and 5 mL for >17 years old) were collected for individuals presenting with history of fever ≥2 days or with axillary temperature of ≥38 °C, and clinical findings were systematically recorded by a study physician. Identity cards distributed during the censuses enabled identification of participants when presenting for febrile illness related care and treatment. For individuals not having the identification cards, computerized censuses on electronic tablets available at each study site were used to confirm identity. Blood specimens for culture were obtained and transported on the same day of collection to laboratories at the icddr,b. Blood cultures were monitored in an automated BacT/ALERT system, and subculturing was done on MacConkey, chocolate, and blood agar plates following a positive signal by the machine. Colony morphology, microscopic examination of Gram stain, biochemical tests, and slide agglutination tests were used for identification of *Salmonella* isolates. *Salmonella*-specific antisera including polyvalent O and O1, serogroup “O” *Salmonella* antigens [D], and flagellar “H” antigens [d] (Denka Sieken) were used for agglutination [16,17]. Treatment for patients with typhoid fever was based on the physicians’ discretion, guided by antimicrobial susceptibility results. A single febrile episode was considered for febrile visits with the onset of fever ≤14 days after discharge for a previous febrile visit. A home visit was performed within 14 days of discharge for all participants with positive blood cultures to confirm that the person whose name was given on the day of presentation to the treatment center had indeed sought treatment on that date. A typhoid fever episode was defined as a febrile episode with a confirmed identity during which at least 1 blood culture yielded *S enterica* serotype Typhi (*Salmonella* Typhi).

### Variable Selection to Define “Better” Versus “Not Better” WASH

As a first step, we partitioned each WASH variable collected during the censuses into a binary variable defining “Better” *versus* “Not Better” household WASH. The variables were partitioned based on substantive judgment aligned with the local context, and without prior knowledge of the typhoid incidence rates in participants with different categories of these variables. We then divided the households of total population in the JE clusters (75 clusters) at random into 2 mutually exclusive subpopulations: 98,087 (60%) of households constituting a “training” subpopulation and the other 65,286 (40%) a “validation” subpopulation. Variables associated with typhoid risk in bivariate Cox regression models at *P*≤.20 in the training subpopulation were selected for model development. Inclusion of all 13 WASH variables regardless of associations of the individual variables with the risk of typhoid, did not reveal any improvement in model performance. Proportionality assumptions for each variable were confirmed before inclusion in the bivariate models.

### Development of the WASH Decision Tree to Predict Typhoid in the Training Subpopulation

As described elsewhere, we developed a composite WASH variable based on the selected candidate WASH variables to predict typhoid risk using a recursive partitioning-based algorithm in the training subpopulation [8,18]. In brief, we developed a binary WASH rule that predicts typhoid fever risk using individual WASH variables that were individually associated with the risk of typhoid fever and then conjoined as specified by the decision tree. Ultimately, the decision tree represents a composite WASH rule based on a combination of these WASH variables (Better versus Not Better WASH) predicting a classification of no typhoid versus typhoid, respectively. The decision tree then yielded several terminal nodes predictive of “higher” or “lower” risk of typhoid fever, which then served as alternative cutoffs used to create a dichotomous composite rule for “higher” or “lower” risk of typhoid, using the Youden index [12]. The WASH features in the decision tree predictive of “higher” risk of typhoid in this dichotomous typhoid variable then defined “Not Better” WASH and the features predictive of “lower” risk of typhoid in the dichotomous variable defined “Better” WASH.

The prediction rule was designed to discriminate the risk of typhoid fever in a dynamic population over the 2-year period of postvaccination follow-up in the trial. The algorithm was first evaluated in the training subpopulation assuming 1:1 ratio for a default loss of function of the cost of false positive and false negative classifications, with at least 300 observations at each terminal node. Subsequently, the algorithm was cross validated with estimation of the cross-validation error in 1 of 10 randomly assembled partitions of the training subpopulation. The 10-fold cross-validation technique was employed with the “*rpart*” package in the R software (R Foundation for Statistical Computing). We first constructed a tree using the training data, including terminal nodes. Then, we pruned the tree to obtain the smallest tree with the lowest misclassification error. The minimal complexity parameter was used to prune the model in finding the optimal prediction rule providing the minimum error with at least 2 terminal nodes in the tree. A receiver operating characteristic (ROC) curve was used to define the AUC for different cutoffs for dichotomizing the predictive rule, with the optimal cutoff for typhoid prediction selected as that which maximized the Youden Index, which includes sensitivity plus specificity, in relation to the proportion of participants in the terminal nodes developing typhoid fever [19].

### Evaluation of the Composite WASH Variable in a Validation Subpopulation and Prediction of Typhoid Risk in the Total Population

To evaluate reproducibility of sensitivity and specificity of the composite WASH variable developed in the training subpopulation, we tested the variable in a distinct *validation* subpopulation. After ascertaining concordance of sensitivity (the proportion of participants developing typhoid fever who lived in households with “Not Better” WASH) and specificity (the proportion of participants not developing typhoid fever who lived in households with “Better” WASH) in predicting typhoid risk between the training and validation subpopulations, we then evaluated the ability of the variable to predict typhoid risk in the total population in the JE clusters.

### Data Analysis

We constructed Cox proportional hazard regression models to evaluate the association between household WASH status and risk of typhoid in household inhabitants as a function of time to the first typhoid episode. Follow-up of persons in the baseline census began at the time of the census, and follow-up of those entering later as births or in-migrants began on the date of entry into the population. Follow-up was right censored by death, outmigration, and the end of this study. Before inclusion in the models, proportionality assumptions for all independent variables were assessed by a bivariate Cox model. The models were adjusted for randomization stratifying variables including geographic ward, longer than median distance to this study’s clinics, and number of eligible children residing in each cluster at baseline. As well, other baseline covariates, including age in years, number of participants in clusters, religion (Muslim), and monthly household expenditure (in Bangladeshi taka), were fitted as independent variables if they were associated with the hazard of typhoid in bivariate Cox models at *P*<.15. The average expenditure of households in a cluster was used as the monthly expenditure of households with unspecified monthly expenditure. The model coefficient for living in a “Better” WASH household was exponentiated to estimate the HR of typhoid fever, and the 95% CI for the HR was estimated using a robust sandwich method to adjust for the design effect of the clusters. Protection by Better WASH against typhoid fever was assessed as [(1 – HR) × 100%)] with corresponding 95% interval limits for percentage protection. We also used Cox models to evaluate the protective association between the cluster prevalence of “Better” WASH households and the risk of typhoid in the total population during the follow-up period. The “Better” WASH cluster prevalence during the 2 years follow-up was calculated as the proportion of PYO contributed by members of households with “Better” WASH in the cluster divided by the total PYO for members of all households in the same cluster. We also assessed the protective association between increasing levels of “Better” WASH cluster prevalence, expressed in Cox models dimensionally, and the risk of typhoid after adjustment for the above-mentioned covariates. In these models, “Better” WASH coverage of the cluster was assigned as the “Better” WASH coverage datapoint for each resident in the respective cluster.

The analysis was performed using the *rpart* package for decision tree modelling, *rpart.plot* package for tree plotting, *pROC* package for the ROC curve, *survival* package for the Cox model, and *dplyr* package for data management under R Studio analytical software (R Foundation for Statistical Computing) [20-22]. The presence of collinearity in the multivariate models was assessed by inspection of variance inflation factors, with high variance inflation factors indicative of collinearity. For all the statistical analyses, a *P*<.05 (2-tailed) was taken as the margin of statistical significance.

### Ethical Considerations

The trial has received ethical approval from the Research and Ethical Review Committees of icddr,b (PR-17115), as well as the institutional review boards of Oxford University and the University of Maryland. Informed written consent from all participants was obtained, with parental or guardian consent for those younger than the age of 16 years, and assent from participants aged 11 years to younger than 16 years. The trial was monitored by a local Data and Safety Monitoring Board established by icddr,b, as well as an international Data and Safety Monitoring Board established by the University of Maryland. This study’s data are anonymous and were securely stored in access-controlled cabinets. The trial has been registered in the ISRCTN Registry under the identifier ISRCTN11643110.

## Results

### Assembly and Characteristics of Training and Validation Subpopulations

**Figure 1:**
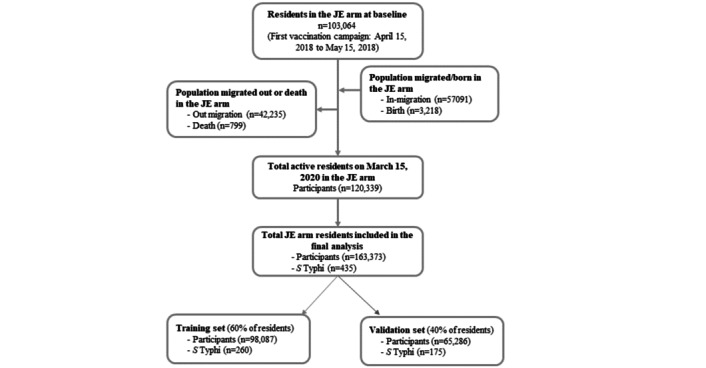
CONSORT (Consolidated Standards of Reporting Trials) diagram of population dynamics during April 15, 2018, and March 15, 2020. JE: Japanese encephalitis; N: number; *S* Typhi: *Salmonella enterica* serovar Typhi.

A total of 103,064 individuals living in 25,478 households within the control clusters for the trial were enumerated during the baseline census. In these households, 51,211 (49.7%) individuals were men, and the mean age was 26.8 (SD 17.3) years. During the 2 years of follow-up, 57,091 persons migrated into this study’s area, 3218 were born, and 43,034 migrated out of this study’s area or died, making the population residing in the JE clusters at the last census 120,339 persons, and the population ever followed in these clusters during the 2 years 163,373 persons (Figure 1). The training subpopulation comprised 98,087 individuals with a mean age 25.9 (SD 17.3) years, while the validation subpopulation included 65,286 individuals with a mean age 25.9 (SD 17.3) years (Table 1). Men were 48,638 (49.6%) in the training subpopulation and 32,496 (49.8%) in the validation subpopulation. During the 2-years of follow-up, a total of 435 first episodes of typhoid cases were detected, yielding an incidence of 209/100,000 PYO in the total population residing in the control clusters. The numbers of typhoid cases in the training and validation subpopulations were 260 and 175, respectively.

**Table 1:** Baseline characteristics of the total, training, and validation populations in the control clusters of the Vi-TT^a^ cluster randomized trial, Dhaka, Bangladesh.

Characteristics	Full set	Training set	Validation set
Age (years), mean (SD)	25.9 (17.3)	25.9 (17.3)	25.9 (17.3)
Gender: men, n (%)	81,134 (49.7)	48,638 (49.6)	32,496 (49.8)
Religion: Muslim, n (%)	161,591 (98.9)	97,004 (98.9)	64,587 (98.9)
Monthly expenditure (BDT^b^)^c^, mean (SD)	17,420.3 (9053.2)	17,397.5 (7793)	17,454.6 (10,670.3)
Toilet facility: private, n (%)	68838 (42.1)	41155 (42)	27683 (42.4)
Adult toilet: flush toilet, n (%)	7804 (4.8)	4617 (4.7)	3187 (4.9)
Child toilet: flush toilet, n (%)	1000 (0.6)	580 (0.6)	420 (0.6)
Source of drinking water: private tap, well, or pump; water vendor, n (%)	63,263 (38.7)	37,854 (38.6)	25,409 (38.9)
Treated drinking water, n (%)	122,258 (74.8)	73,527 (75)	48,731 (74.6)
Treated cleaning water, n (%)	475 (0.3)	300 (0.3)	175 (0.3)
WASH^d^ before meals, n (%)	121,595 (74.4)	73,019 (74.4)	48,576 (74.4)
Hand wash after defecation, n (%)	159,390 (97.6)	95,698 (97.6)	63,692 (97.6)
Handwashing water available in HH^e^, n (%)	159,193 (97.5)	95,616 (97.5)	63,577 (97.4)
Handwashing soap available in HH, n (%)	161,443 (98.8)	96,985 (98.9)	64,458 (98.7)
Waste disposal place: fixed disposal, n (%)	155,458 (95.2)	93,406 (95.2)	62,052 (95)
Distance to drinking water source: shorter than median distance, n (%)	108,129 (66.2)	64,772 (66)	43,357 (66.4)
Water filter available in HH, n (%)	19,271 (11.8)	11,628 (11.9)	7643 (11.7)

^a^Vi-TT: Vi-tetanus toxoid.

^b^BDT: Bangladeshi taka; currency conversion rate: US $1 = BDT 110.48

^c^In total, 75 households had missing household expenditures, which were then substituted with the average household expenditure.

^d^WASH: waster, sanitation, and hygiene.

^e^HH: household.

### Model Development, Training, and Validation

**Figure 2:**
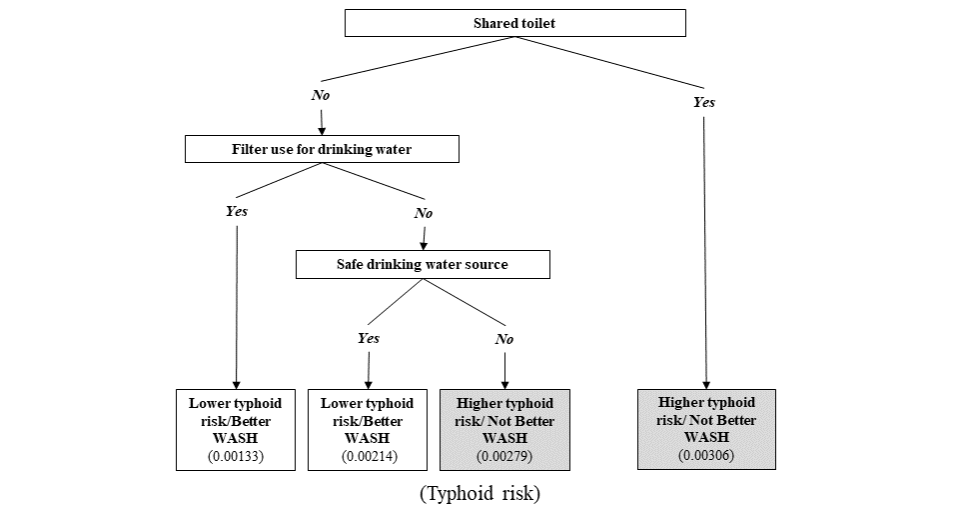
Decision tree defining “Better” and “Not Better” household WASH using 3 binary WASH variables collected at first census. The figures within the parentheses in the final boxes of the decision tree indicate the proportion of individuals who tested positive for typhoid fever. Out of 40,329 households, 18 (0.0446%) experienced 2 typhoid episodes. Among these, 13 out of 28,230 (0.046%) were from households categorized as Not Better WASH, while 5 out of 12,099 (0.0413%) were from households categorized as Better WASH. WASH: water, sanitation, and hygiene.

In the training subpopulation, having a private toilet (HR 0.57, 95% CI 0.43-0.77; *P*<.001); a safe source of drinking water, defined as a private tap, private well, bottled water, or water vendor (HR 0.65, 95% CI 0.49-0.87; *P*=.003); and availability of a water filter in the household during observation (HR 0.73, 95% CI 0.45-1.18; *P*=.20) were associated with typhoid risk among members of the household (Table 2). These 3 variables were used to build a binary composite prediction rule defining “Better” *versus* “Not Better” household WASH using the recursive partitioning algorithm (Figure 2).

**Table 2:** Bivariate associations of binary WASH^a^ variables with typhoid risk in the training subpopulation of the control clusters of the Vi-TT^b^ cluster randomized trial, Dhaka, Bangladesh.

Variables	Yes					No					Hazard ratio (HR^c^)^d^	
	n	Cases	PY^e^	IR^f^/100,000 PY	n		Cases	PY	IR/100,000 PY	HR (95% CI)^g^		*P* value^g^
Toilet facility: private	41,155	86	57,718	149	56,932		174	67,381	258	0.57 (0.43-0.77)		<.001
Adult toilet: flush toilet	4617	11	6223	177	93,470		249	11,8876	209	1.07 (0.35-3.30)		.91
Child toilet: flush toilet	580	2	749	267	97,507		258	124,350	207	1.65 (0.41-6.69)		.48
Source of drinking water: private tap, well, or pump; water vendor	37,854	82	52,216	157	60,233		178	72,882	244	0.65 (0.49-0.87)		.003
Treated drinking water	73,527	183	92,350	198	24,560		77	32,748	235	0.91 (0.65-1.27)		.57
Treated cleaning water	300	1	420	238	97,787		259	124,678	208	1.13 (0.22-5.78)		.89
Hand wash before meals	73,019	191	92,584	206	25,068		69	32,514	212	1.15 (0.87-1.52)		.33
Hand wash after defecation	95,698	251	122,048	206	2389		9	3051	295	0.82 (0.48-1.40)		.47
Handwashing water available in HH^h^	95,616	251	121,736	206	2424		9	3312	272	0.94 (0.33-2.70)		.91
Handwashing soap available in HH	96,985	257	123,590	208	1102		3	1508	199	1.3 (0.56-3.03)		.537
Waste disposal place: fixed disposal	93,406	240	118,789	202	4681		20	6309	317	0.77 (0.43-1.37)		.37
Distance to drinking water source: shorter than median distance	64,772	175	83,175	210	33,315		85	41,923	203	1.11 (0.85-1.45)		.46
Water filter available in HH	11,628	24	15,966	150	86,459		236	109,132	216	0.73 (0.45-1.18)		.20

^a^WASH: water, sanitation, and hygiene.

^b^Vi-TT: Vi-tetanus toxoid.

^c^HR: hazard ratio.

^d^Estimated from extended Cox proportional hazards model.

^e^PY: person years.

^f^IR: incidence rate.

^g^Calculated using robust SE assuming risk of typhoid is correlated within clusters.

^h^HH: household.

### Performance of the Composite WASH Variable Predicting Typhoid Fever

**Figure 3:**
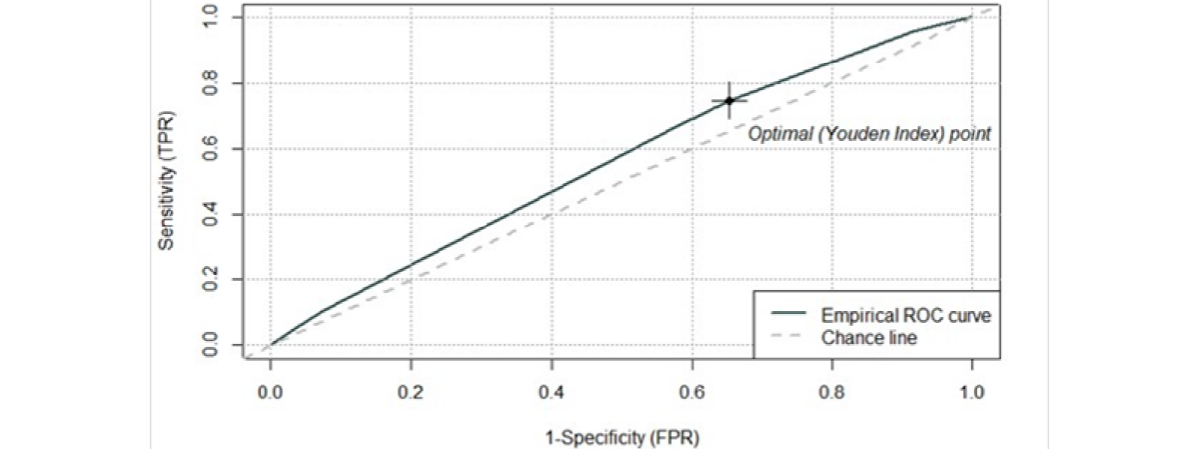
Receiver operator characteristic curve used to define the best cut off value to classify households as having “Better” versus “Not Better” WASH. AUC=56% (95% CI 53-59); best cutoff using the Youden index is 0.0025. AUC: area under the receiver operator characteristic curve; FPR: false positive rate; ROC: receiver operator characteristic; TPR, true positive rate; WASH: water, sanitation, and hygiene.

The decision tree is shown in Figure 2. The dominant bifurcation for the prediction of typhoid in the tree was having a private toilet. In the training subpopulation, an optimal cutoff value of 0.0025 maximized the Youden index using the ROC curve, with an AUC of 56% (95% CI 53-59; Figure 3). With this threshold, the rule had a sensitivity of 74.6% (95% CI 68.9-80.0) and specificity of 34.7% (95% CI 34.4-35.0). Applying this rule to the validation subpopulation yielded comparable characteristics to those observed in the training subpopulation with a sensitivity and specificity of 74.9% (95% CI 67.8-81.1) and 35.2% (95% CI 34.9-35.6), respectively. The positive and negative predictive values were 0.3% (95% CI 0.26-0.35) and 99.8% (95% CI 99.7-99.9), respectively.

### Prediction of Typhoid Incidence by Household WASH Status in the Total Population

We next applied the WASH prediction rule to the overall population residing in the control clusters to predict typhoid risk. The incidence of typhoid fever in all age groups living in “Better” WASH households was 139/100,000 PYO compared to 252/100,000 PYO in those living in “Not Better” WASH households, a reduction in typhoid risk of 38% (95% CI 22-51; *P*<.001) after adjusting for covariates. The reduction in typhoid risk was largely attributed to the protection associated with living in “Better” WASH households among children <10 years old (adjusted hazard ratio [aHR] 0.49, 95% CI 0.36-0.66; *P*<.001). Protective associations were aHR 0.35 (95% CI 0.20-0.61; *P*<.001) in children 2-4 years old, and aHR 0.58 (95% CI 0.36-0.92; *P*=.02) in 5-9 years old (Table 3 and Figure S1 in Multimedia Appendix 1).

**Figure 4:**
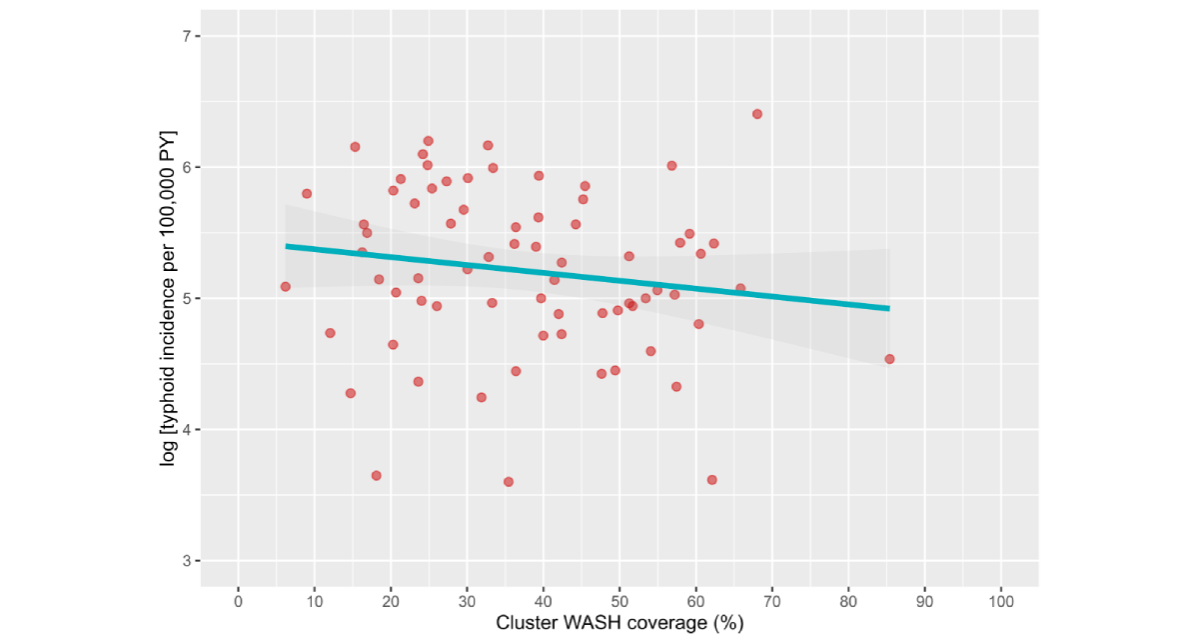
Increasing levels of “Better” WASH cluster prevalence and the risk of typhoid in the total population residing in the control clusters of the Vi-TT cluster randomized trial. Out of 40,329 households, 28,230 (70%) were categorized as “Not Better” WASH households, and the remaining 12,099 (30%) fell under the “Better” WASH category. PY: person years; Vi-TT: Vi-tetanus toxoid; WASH: water, sanitation, and hygiene.

The level of “Better” WASH cluster prevalence appeared inversely related to the log incidence of typhoid (Figure 4). However, the association between an increasing prevalence of “Better” WASH households in a cluster and typhoid risk was not significant in the adjusted multivariable model (aHR 0.996; 95% CI 0.986-1.006; *P*=.39) for each percent increase in the prevalence of “Better WASH” households in the cluster. The linear “Better” WASH cluster prevalence exhibited collinearity with household WASH status in the multivariable model, which precluded simultaneous estimation of the protective effects associated with living in “Better” WASH households and those associated with living in clusters with increasing prevalence of “Better” WASH households.

**Table 3:** Individual-level protection against typhoid associated with “Better” household WASH^a^ in the total population residing in the control clusters of the Vi-TT^b^ cluster randomized trial, Dhaka, Bangladesh, stratified by age at onset of follow-up.

Years	“Better” WASH					“Not Better” WASH					Hazard ratio (HR^c^)^d^			
	n^e^	Cases	PY^f^	IR^g^/100,000 PY	N		Cases	PY	IR/100,000 PY	Crude HR^h^		*P* value^h^	Adjusted HR^h,i^	*P* value^h^
All	56,969	110	79,326	139	106,404		325	129,088	252	0.56 (0.44,0.71)		<.001	0.62 (0.49-0.78)	<.001
<2 years	3356	13	3883	335	6979		45	7211	624	0.54 (0.28,1.06)		.08	0.65 (0.32-1.28)	.21
2-4 years	3131	18	4424	407	6438		93	7919	1174	0.35 (0.22,0.57)		<.001	0.35 (0.20-0.61)	<.001
5-9 years	4894	26	7086	367	9819		84	12,586	667	0.57 (0.35,0.94)		.03	0.58 (0.36-0.92)	.02
10-14 years	4997	20	7278	275	9265		38	12,123	313	0.88 (0.5,1.56)		.66	0.92 (0.53-1.59)	.77
>15 years	40,591	33	56,655	58	73,903		65	89,250	73	0.76 (0.48,1.21)		.25	0.79 (0.50-1.24)	.31

^a^WASH: water, sanitation, and hygiene.

^b^Vi-TT: Vi-tetanus toxoid.

^c^HR: hazard ratio.

^d^Hazard ratios are estimated from extended Cox proportional hazards model.

^e^n: number; 100K: 100,000.

^f^PY: person years.

^g^IR: incidence rate.

^h^*P* values and CI are calculated using robust SE assuming risk of typhoid is correlated within clusters.

^i^Hazard ratio adjusted for the stratifying variables for randomization, including geographic ward, longer distance to study clinics than median distance, number of eligible children at baseline, and other baseline covariates age, Muslim religion; total monthly income; number of participants in a cluster.

## Discussion

We developed a composite WASH variable for a poor urban population in Dhaka using a machine learning approach, where 3 binary WASH variables associated with typhoid risk were combined to discriminate “Better” *versus* “Not Better” household WASH status. The variable predicted the risk of typhoid fever after adjusting for confounding variables including household socioeconomic status, with individuals in households with “Better” WASH having a 38% lower risk of typhoid than those in households with “Not Better” WASH, a reduction in risk that appeared primarily attributable to reduced risk in children younger than the age of 10 years. The higher risk reduction in the younger age group with improved WASH practices can be potentially explained by the elevated incidence of typhoid among this demographic [23,24]. Further, there may be other behavioral disparities between adults and children, such as children primarily staying at home and adults being more frequently outdoors for work-related activities, which could contribute to this discrepancy.

The WASH variables defining the composite household WASH rule in this analysis are somewhat different from those found to predict the risk of typhoid in an earlier analysis in Kolkata [8], except for quality of drinking water, which contributed to the definition of the composite WASH variable in both analyses. These differences are, however, anticipated owing to the differences between the 2 settings including sociocultural characteristics, data collection time, and the different data instruments used for characterizing WASH in the 2 studies. Furthermore, both studies were primarily designed to evaluate typhoid vaccine protection, and neither study attempted in-depth characterization of WASH with the data collection instruments, so component criteria of the composite variables are very likely to be markers of WASH facilities and practices, rather than the specific facilities and practices per se that account for a reduced risk of typhoid. However, associations with typhoid in both settings were independent of socioeconomic status, underscoring that it was likely that better WASH rather than greater affluence was responsible for the protective associations with typhoid.

Some limitations should be noted while interpreting the results. First, as noted earlier, we used WASH variables collected as part of the randomized clinical trial of vaccines and were not ascertained for determination of the balance of the randomized intervention arms rather than as in a specific study of WASH. Second, our initial dichotomization of WASH variables in the population was based on perceived clinical and public health significance, although such judgements were made without knowledge of typhoid outcomes. Third, our creation of a composite WASH variable for households in a dichotomous fashion was done to create a simplified assessment of household level WASH and cluster prevalence of “Better” WASH, but this could have led to a potential loss of information on certain WASH variables, which might make our analysis conservative. An additional factor potentially influencing data validity is the Hawthorne effect, arising from alterations in observed behaviors.

Despite these limitations, our analysis has several strengths. First, typhoid incidence data used in this analysis were collected using a prospective cohort with comprehensive surveillance of typhoid fever with repeated censuses and WASH assessments that allowed analysis of a dynamic cohort in a highly mobile population. The intensified community engagement and proactive house-to-house visits by community health workers have played a role in ensuring that the proportion of participants across all age groups who refrained from seeking care for febrile episodes did not result in notable variations in care-seeking practices. Second, the detailed demarcation of the clusters using natural structures, when possible, enabled unambiguous estimates of the “Better” WASH coverage in the clusters [6]. Third, external validation of the WASH prediction rule using a separate validation subpopulation revealed similar levels of sensitivity and specificity, suggesting that the prediction of typhoid by the composite variable was not exaggerated by overfitting the rule to the data used for derivation.

Simplified prediction rules, like the one presented in our study, offer great potential for various stakeholders involved in combating typhoid fever, including public health decision makers, researchers, and clinicians. These rules highlight the effectiveness of existing WASH adaptations, which can be implemented by most urban slum households to reduce susceptibility to typhoid in such environments. They can also be used to guide vaccine roll-out in high-risk populations and for community engagement initiatives aimed at increasing awareness of typhoid risk. Since the WASH data was collected from a diverse urban slum in Dhaka, the results can be extrapolated to other slums in Dhaka and similar regions with high typhoid burdens. Although further investigation is needed to address implementation barriers of “Better” WASH parameters, well-designed risk communication strategies can facilitate the adoption of such adaptations [25]. While not directly related to the clinical diagnosis and treatment of individual typhoid cases, WASH interventions play a crucial role in the broader strategy by potentially reducing antibiotic resistance. By decreasing antibiotic usage, these interventions contribute to preserving the efficacy of antibiotics, thus expanding the range of treatment options available for combating typhoid fever.

In conclusion, our findings suggest that improvements in WASH that already exist in this slum population without specific external intervention are associated with a reduced risk of typhoid and lend optimism to the notion that simple and inexpensive improvements of WASH to protect against typhoid are possible. Research to develop and evaluate such interventions should be pursued. As well, it will be important to assess how better existing WASH interacted with and perhaps modified protection by Vi-TT typhoid vaccine tested in this CRT. We are carrying out these analyses and will report them in future publications.

## Appendix

Multimedia Appendix 1 Kaplan-Meier curve displaying time to onset of typhoid cases in Better versus Not Better WASH households.

## Abbreviations

aHR: adjusted hazard ratio
AUC: area under the curve
CRT: cluster randomized trial
HR: hazard ratio
JE: Japanese encephalitis
PYO: person years of observation
ROC: receiver operating characteristic
TCV: typhoid conjugate vaccine
Vi-TT: Vi-tetanus toxoid
WASH: water, sanitation, and hygiene

## Data Availability

The data and code will be available upon reasonable request to the corresponding author.

## References

[1] GBD 2017 Typhoid and Paratyphoid Collaborators (2019). The global burden of typhoid and paratyphoid fevers: a systematic analysis for the Global Burden of Disease Study 2017. Lancet Infect Dis.

[2] Parry CM, Hien TT, Dougan G, White NJ, Farrar JJ (2002). Typhoid fever. N Engl J Med.

[3] (2023). Typhoid. World Health Organization.

[4] Marchello CS, Birkhold M, Crump JA (2020). Complications and mortality of typhoid fever: a global systematic review and meta-analysis. J Infect.

[5] Fatima M, Kumar S, Hussain M, Memon NM, Vighio A, Syed MA, Chaudhry A, Hussain Z, Baig ZI, Baig MA, Asghar RJ, Ikram A, Khader Y (2021). Morbidity and mortality associated with typhoid fever among hospitalized patients in hyderabad district, Pakistan, 2017-2018: retrospective record review. JMIR Public Health Surveill.

[6] Vighio A, Syed MA, Hussain I, Zia SM, Fatima M, Masood N, Chaudry A, Hussain Z, Iqbal Baig MZ, Baig MA, Ikram A, Khader YS (2021). Risk factors of extensively drug resistant typhoid fever among children in Karachi: case-control study. JMIR Public Health Surveill.

[7] Stanaway JD, Atuhebwe PL, Luby SP, Crump JA (2020). Assessing the feasibility of typhoid elimination. Clin Infect Dis.

[8] Im J, Islam MT, Ahmmed F, Kim DR, Khan AI, Zaman K, Ali M, Marks F, Qadri F, Kim J, Clemens JD (2021). Can existing improvements of water, sanitation, and hygiene (WASH) in urban slums reduce the burden of typhoid fever in these settings?. Clin Infect Dis.

[9] Brockett S, Wolfe MK, Hamot A, Appiah GD, Mintz ED, Lantagne D (2020). Associations among water, sanitation, and hygiene, and food exposures and typhoid fever in case-control studies: a systematic review and meta-analysis. Am J Trop Med Hyg.

[10] Breiman RF, Cosmas L, Njuguna H, Audi A, Olack B, Ochieng JB, Wamola N, Bigogo GM, Awiti G, Tabu CW, Burke H, Williamson J, Oundo JO, Mintz ED, Feikin DR (2012). Population-based incidence of typhoid fever in an urban informal settlement and a rural area in Kenya: implications for typhoid vaccine use in Africa. PLoS One.

[11] Mogasale VV, Ramani E, Mogasale V, Park JY, Wierzba TF (2018). Estimating typhoid fever risk associated with lack of access to safe water: a systematic literature review. J Environ Public Health.

[12] Luby SP (2018). Urban slums: a supportive ecosystem for typhoidal salmonellae. J Infect Dis.

[13] Akullian A, Ng'eno E, Matheson AI, Cosmas L, Macharia D, Fields B, Bigogo G, Mugoh M, John-Stewart G, Walson JL, Wakefield J, Montgomery JM (2015). Environmental transmission of typhoid fever in an urban slum. PLoS Negl Trop Dis.

[14] Naheed A, Ram PK, Brooks WA, Hossain MA, Parsons MB, Talukder KA, Mintz E, Luby S, Breiman RF (2010). Burden of typhoid and paratyphoid fever in a densely populated urban community, Dhaka, Bangladesh. Int J Infect Dis.

[15] Theiss-Nyland K, Qadri F, Colin-Jones R, Zaman K, Khanam F, Liu X, Voysey M, Khan A, Hasan N, Ashher F, Farooq YG, Pollard AJ, Clemens JD (2019). Assessing the impact of a Vi-polysaccharide conjugate vaccine in preventing typhoid infection among Bangladeshi children: a protocol for a phase IIIb trial. Clin Infect Dis.

[16] Khanam F, Sheikh A, Sayeed MA, Bhuiyan MS, Choudhury FK, Salma U, Pervin S, Sultana T, Ahmed D, Goswami D, Hossain ML, Mamun KZ, Charles RC, Brooks WA, Calderwood SB, Cravioto A, Ryan ET, Qadri F (2013). Evaluation of a typhoid/paratyphoid diagnostic assay (TPTest) detecting anti-Salmonella IgA in secretions of peripheral blood lymphocytes in patients in Dhaka, Bangladesh. PLoS Negl Trop Dis.

[17] Khanam F, Sayeed MA, Choudhury FK, Sheikh A, Ahmed D, Goswami D, Hossain ML, Brooks A, Calderwood SB, Charles RC, Cravioto A, Ryan ET, Qadri F (2015). Typhoid fever in young children in Bangladesh: clinical findings, antibiotic susceptibility pattern and immune responses. PLoS Negl Trop Dis.

[18] Strobl C, Malley J, Tutz G (2009). An introduction to recursive partitioning: rationale, application, and characteristics of classification and regression trees, bagging, and random forests. Psychol Methods.

[19] Zou KH, O'Malley AJ, Mauri L (2007). Receiver-operating characteristic analysis for evaluating diagnostic tests and predictive models. Circulation.

[20] Robin X, Turck N, Hainard A, Tiberti N, Lisacek F, Sanchez JC, Müller M (2011). pROC: an open-source package for R and S+ to analyze and compare ROC curves. BMC Bioinformatics.

[21] Therneau TM, Grambsch PM (2000). Modeling Survival Data: Extending the Cox Model.

[22] Wickham H, François R, Henry L, Müller K, Vaughan D, Posit Software, PBC (2018). dplyr: a grammar of data manipulation. R package version 0.7.6.

[23] Garrett DO, Longley AT, Aiemjoy K, Yousafzai MT, Hemlock C, Yu AT, Vaidya K, Tamrakar D, Saha S, Bogoch II, Date K, Saha SK, Islam MS, Sayeed KMI, Bern C, Shakoor S, Dehraj IF, Mehmood J, Sajib MSI, Islam M, Thobani RS, Hotwani A, Rahman N, Irfan S, Naga SR, Memon AM, Pradhan S, Iqbal K, Shrestha R, Rahman H, Hasan MM, Qazi SH, Kazi AM, Saddal NS, Jamal R, Hunzai MJ, Hossain T, Marks F, Carter AS, Seidman JC, Qamar FN, Saha SK, Andrews JR, Luby SP (2022). Incidence of typhoid and paratyphoid fever in Bangladesh, Nepal, and Pakistan: results of the Surveillance for enteric fever in Asia project. Lancet Glob Health.

[24] Marks F, von Kalckreuth V, Aaby P, Adu-Sarkodie Y, El Tayeb MA, Ali M, Aseffa A, Baker S, Biggs HM, Bjerregaard-Andersen M, Breiman RF, Campbell JI, Cosmas L, Crump JA, Espinoza LMC, Deerin JF, Dekker DM, Fields BS, Gasmelseed N, Hertz JT, Van Minh Hoang N, Im J, Jaeger A, Jeon HJ, Kabore LP, Keddy KH, Konings F, Krumkamp R, Ley B, Løfberg SV, May J, Meyer CG, Mintz ED, Montgomery JM, Niang AA, Nichols C, Olack B, Pak GD, Panzner U, Park JK, Park SE, Rabezanahary H, Rakotozandrindrainy R, Raminosoa TM, Razafindrabe TJL, Sampo E, Schütt-Gerowitt H, Sow AG, Sarpong N, Seo HJ, Sooka A, Soura AB, Tall A, Teferi M, Thriemer K, Warren MR, Yeshitela B, Clemens JD, Wierzba TF (2017). Incidence of invasive salmonella disease in sub-Saharan Africa: a multicentre population-based surveillance study. Lancet Glob Health.

[25] Barac R, Als D, Radhakrishnan A, Gaffey MF, Bhutta ZA, Barwick M (2018). Implementation of interventions for the control of typhoid fever in low- and middle-income countries. Am J Trop Med Hyg.

