# Supplementary figures and images for: Association Among Household Water, Sanitation, and Hygiene (WASH) Status and Typhoid Risk in Urban Slums: Prospective Cohort Study in Bangladesh
Source: JMIR Public Health Surveill. 2023 Nov 20;9:e41207. doi: 10.2196/41207 (PMC10696503; doi:10.2196/41207)

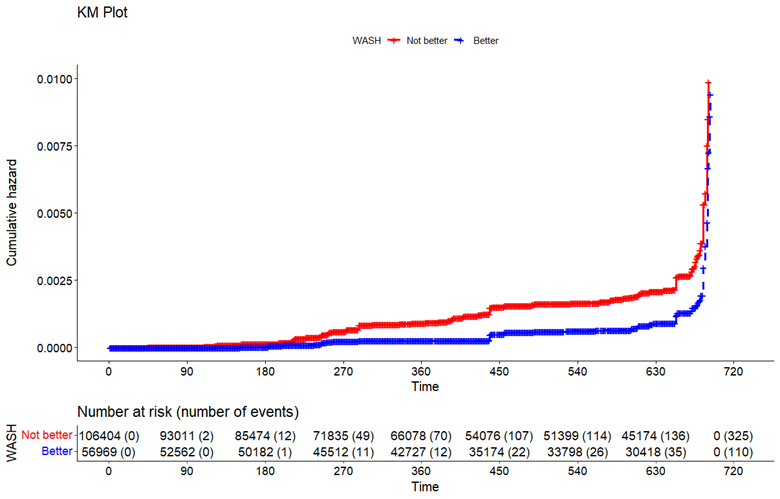

Supplement: Multimedia Appendix 1 [file publichealth_v9i1e41207_app1.png]
